# Youth mental health first aid: a description of the program and an initial evaluation

**DOI:** 10.1186/1752-4458-5-4

**Published:** 2011-01-27

**Authors:** Claire M Kelly, Johanna M Mithen, Julie A Fischer, Betty A Kitchener, Anthony F Jorm, Adrian Lowe, Chris Scanlan

**Affiliations:** 1 Orygen Youth Health Research Centre, Centre for Youth Mental Health, University of Melbourne, Parkville, VIC, Australia; 2 School of Population Health, University of Melbourne, Parkville, VIC, Australia

## Abstract

**Background:**

Adolescence is the peak age of onset for mental illness, with half of all people who will ever have a mental illness experiencing their first episode prior to 18 years of age. Early onset of mental illness is a significant predictor for future episodes. However, adolescents and young adults are less likely than the population as a whole to either seek or receive treatment for a mental illness. The knowledge and attitudes of the adults in an adolescent's life may affect whether or not help is sought, and how quickly. In 2007, the Youth Mental Health First Aid Program was launched in Australia with the aim to teach adults, who work with or care for adolescents, the skills needed to recognise the early signs of mental illness, identify potential mental health-related crises, and assist adolescents to get the help they need as early as possible. This paper provides a description of the program, some initial evaluation and an outline of future directions.

**Methods:**

The program was evaluated in two ways. The first was an uncontrolled trial with 246 adult members of the Australian public, who completed questionnaires immediately before attending the 14 hour course, one month later and six months later. Outcome measures were: recognition of schizophrenia or depression; intention to offer and confidence in offering assistance; stigmatising attitudes; knowledge about adolescent mental health problems and also about the Mental Health First Aid action plan. The second method of evaluation was to track the uptake of the program, including the number of instructors trained across Australia to deliver the course, the number of courses they delivered, and the uptake of the YMHFA Program in other countries.

**Results:**

The uncontrolled trial found improvements in: recognition of schizophrenia; confidence in offering help; stigmatising attitudes; knowledge about adolescent mental health problems and application of the Mental Health First Aid action plan. Most results were maintained at follow-up. Over the first 3 years of this program, a total of 318 instructors were trained to deliver the course and these instructors have delivered courses to 10,686 people across all states and territories in Australia. The program has also spread to Canada, Singapore and England, and will spread to Hong Kong, Sweden and China in the near future.

**Conclusions:**

Initial evaluation suggests that the Youth Mental Health First Aid course improves participants' knowledge, attitudes and helping behaviour. The program has spread successfully both nationally and internationally.

**Trial registration:**

ACTRN12609000033246

## Background

Mental illness is common, but many people do not seek professional help for such problems [1]. An improvement in mental health literacy of the public may result in more people being encouraged to seek professional help and in increased support by family and friends when mental health problems are apparent. The Mental Health First Aid (MHFA) Training and Research Program has developed training courses to improve the mental health literacy of members of the public [2-4].

Adolescence is the peak age of onset for mental illness [5]. The Youth Mental Health First Aid (YMHFA) course is a specialty variant on the standard MHFA course and is designed to improve the mental health literacy of adults who assist adolescents.

### The Standard Mental Health First Aid course

The 12-hour standard MHFA course was launched in 2001. The course is designed to teach members of the public how to support someone who might be developing a mental health problem or experiencing a mental health-related crisis, and to assist them to receive professional help and other supports. Each participant receives a course manual and a certificate of attendance [6].

The course provides an overview of mental health problems, the signs and symptoms of individual mental health problems, the risk factors for specific mental illnesses and available evidence-based treatments. The illnesses covered are depressive, anxiety, psychotic and substance use disorders.

The course has a strong focus on skills and provides a Mental Health First Aid Action Plan. The actions are not intended to be taken in a specific order. The Action Plan comprises five actions, forming the acronym 'ALGEE':

• Assess the risk of suicide or harm

• Listen non-judgmentally

• Give reassurance and information

• Encourage the person to get appropriate professional help

• Encourage self-help strategies

Participants are taught how to apply the Action Plan to help people with the mental health problems described above. In addition, a number of crisis situations are covered; suicide, acute psychosis, excessive use of alcohol, drug overdose, perceived aggressive behaviour, panic attacks and traumatic events.

Evaluation trials [2-4,7,8] have shown that participation in the course increases knowledge, reduces stigma, increases confidence in offering appropriate help, and improves actions taken to help people with mental health problems.

### The Youth Mental Health First Aid course

The 14-hour YMHFA course, launched in 2007, teaches adults how to support adolescents who might be developing a mental health problem or in a mental health crisis, and to assist them to receive professional help. The course content and manual are modified to provide information which is specific to adolescents [9]. In addition to the mental health problems covered in the standard course, YMHFA covers eating disorders. There is also information provided about how to assist a young person who has been engaging in non-suicidal self-injury. There is a strong theme throughout the whole program about the importance of early intervention to minimise the impact of mental health problems on adolescent development. The course can be delivered flexibly as either two full days (which do not have to be consecutive) or over four sessions of 3.5 hours each.

Targets for the training include parents, school professionals, adults involved in recreational activities with adolescents (e.g., sport coaches and scout leaders) and other adults who work with or care about adolescents. One small trial conducted externally with sports coaches showed promising results [10]. The Action Plan does not differ from that provided in the standard course, although the application is tailored to the needs of adolescents.

### YMHFA Instructor training and program dissemination

Instructors are trained by the YMHFA co-ordinator (CMK) and a consultant trainer, and are supported by an administration team. Instructor training courses are run periodically across Australia, or organisations can arrange for in-house training in order to have a number of instructors available to them.

Instructors in the YMHFA course are not employed by the Program. Rather, successful applicants pay to attend instructor training which equips them to run the course, and the Program provides them with ongoing support. Some are funded by their employers to attend instructor training and to run the courses. Others pay their own fees for instructor training, and may offer training on a fee-for-service basis in the community or by arrangement with other organisations.

Instructors who are already accredited to run the standard MHFA program and meet the additional requirements to become YMHFA instructors attend a three day training program. New instructors attend a five and a half day training program. Applicants must have a strong background in youth mental health, experience in running training programs, positive attitudes towards adolescents with mental health problems and either organisational support or a good business plan. Instructors purchase manuals and certificates for use in their own courses. In order to remain accredited to teach the course, a minimum of three courses must be run each year and registered on a secure website.

There is a MHFA website which lists instructors who can be contacted to run courses, and enables them to advertise courses that are open to the public [11]. The website identifies which of the MHFA courses each instructor runs, and identifies very experienced instructors who have run at least 30 courses as Master Instructors. The website also provides information on MHFA, and members of the public can access information they can use if they are unable to attend a course.

### YMHFA Program evaluation

There have been two components to the evaluation of the YMHFA course. The first was an uncontrolled trial with the public, and is described in this paper. The second involves monitoring the uptake and dissemination of the course in Australia and internationally, and is also reported here. In addition, there has been a cluster randomised controlled trial of an abbreviated and modified version of the course adapted for use in schools, which is published elsewhere [12].

## Methods

### Uncontrolled trial

#### Participants

Participants were 246 adults from the general community within two regions of Victoria, Australia who volunteered to attend a 14-hour Youth Mental Health First Aid (YMHFA) course were eligible candidates. Participants were recruited through public advertising and press releases in local newspapers and media outlets and through a local youth mental health service. Seventeen training courses were conducted by one instructor (CS) between October 2007 and May 2008.

#### Measures

Three questionnaires were administered (pre-test, post-test and 6-month follow-up) and are included as Additional File 1. In only the pre-test questionnaire, participants were asked about sociodemographic characteristics, reasons for doing the course, any previous mental health training and history of personal and family mental health problems.

The questionnaire was based on a mental health literacy survey reported previously [13,14]. Participants were presented with two vignettes of a 15 year old, one portraying major depression (Jenny) and one portraying schizophrenia (John). They were given the open-ended question "What, if anything, do you think is wrong with Jenny/John?" Open-ended responses were classified into categories based on coding rules used in a recent study [15]. Multiple responses were allowed. Scoring was conducted by two researchers who rated responses individually and later arrived at a consensus score for each response. Kappa coefficients for inter-rater reliability were computed for both vignettes.

First aid intentions were measured by open-ended questions asking participants what they would do to help each of the young people portrayed in the vignettes. For scoring purposes, a checklist was developed, based on the Mental Health First Aid Action Plan [4]. It incorporates the 5 basic actions described by the acronym 'ALGEE'. Responses were scored out of a possible total of 10 against the checklist, using a 3 point scale (0 = no mention or inadequate response, 1 = superficial response without details, 2 = specific details/actions). If a response contained the word "ALGEE", but nothing else, 1 point was given per action, i.e. total of 5 points. Extra points were given only where specific detail was given for an action. One person scored all the responses. However, to ensure inter-rater reliability, a random sample of 60 responses was independently scored by three other researchers, who later arrived at a consensus.

Confidence in providing first aid was measured by asking participants "How confident would you feel in helping Jenny/John?" Confidence was rated on a 5-point Likert scale ranging from 1 ('not at all') to 5 ('extremely') for each of the vignettes [15].

Stigmatising attitudes were measured by a Personal Stigma scale and a Perceived Stigma scale [16]. Scales were modified to suit attitudes towards adolescents rather than towards adults [17]. In Personal Stigma, the respondent was asked about their own attitudes towards the person described in each vignette while in the Perceived Stigma scale respondents were asked what they thought other people's attitudes were towards each person in the vignette.

Knowledge of mental disorders was measured by a 21 item true/false questionnaire specifically designed to cover information in the course. Response options for each item were 'agree', 'disagree' or 'don't know'. Scoring was based on 1 point per correct response, with 'don't know' being counted as incorrect.

Mental health first aid actions taken were assessed by asking how often a participant has talked to a young person about a mental health problem in the past 6 months. If they had talked with someone, the participant was asked to check the actions they took from a list of 9 options as follows: 1) Spent time listening to their problem; 2) Helped to calm them down; 3) Talked to them about suicidal thoughts; 4) Recommended they seek professional help; 5) Recommended self-help strategies; 6) Gave them information about their problem; 7) Gave them information about local services; 8) Made an appointment for them with services; and 9) Referred them to books or websites about their problem. An additional 'other' category was included for participants to provide details of any other actions undertaken, which were not included in the list. Scoring was based on 1 point per action taken.

The post-test and follow-up questionnaires were the same as the pre-test questionnaire except that both omitted the sociodemographic questions and the questions about actions taken were omitted from the post-test questionnaire.

Questionnaires were given out to participants prior to the commencement of training in the first session. On immediate completion of the training course a post-test questionnaire was given out. Six months thereafter, a follow-up questionnaire was sent to participants by post. Participants who did not submit their 6-month follow-up questionnaire within two weeks of it being sent were posted another follow-up questionnaire with a reminder letter. Where no response was received within a further two week period, a phone call was made to participants, encouraging them to complete the questionnaire and return it at their earliest convenience. A third follow-up questionnaire was posted to participants who had indicated that they had changed address and not received the questionnaire.

#### Ethics

Ethics approval for the trial was granted on 12 July 2007 by The University of Melbourne Health Sciences Human Ethics Sub-Committee (ID: 0714470). There were no adverse events reported.

#### Statistical analysis

Participant responses were entered into a Microsoft Access database (version 2003) with values programmed within a set range to reduce data entry error. Data cleaning and statistical analysis was carried out using STATA (version 10).

Differences between pre-test and post-test on continuous outcome measures were tested using a paired t-test where the distribution was approximately normal, and a sign test where the distribution was clearly non-normal. Differences between pre-test and post-test on dichotomous outcome measures were tested with McNemar's chi-square test.

Because there were missing data at the 6-month time point, logistic regression was carried out to investigate predictors of missing data at 6 months. Complete case analysis was then carried out comparing participants with data at pre-test and 6-month follow-up. Changes were analysed using McNemar's chi-square and sign tests. The P < 0.05 level of significance was used throughout.

Responses were entered into a Microsoft Access database (version 2003) with values programmed within a set range to reduce data entry error and analysed in STATA (version 10).

### Data on program dissemination

Instructor details are recorded on a database contained within the Mental Health First Aid website. Instructors record the courses they run on this database as well. Details recorded include course location and the number of participants who attended. The results of the YMHFA program dissemination were extracted from this database, based on all instructors trained from April 2007, and includes details up to June 20^th^, 2010.

## Results

### Uncontrolled trial

#### Participant characteristics and retention

The participants comprised 58 males and 188 females (mean age of 31 years, range 17 - 68). Fifty percent of participants reported that they had personally experienced a mental health problem, 72% reported that someone in their family had experienced a mental health problem, and 77% reported that they had spoken to a young person about a mental health problem at least once in the previous six months.

Of the 246 participants at pre-test, 221 completed the post-training questionnaire and 138 completed the 6-month follow-up questionnaire. Regression analysis was used to investigate predictors of missing data. At 6-months follow-up, questionnaire data was less likely to be 'missing' for participants who were female; aged 50-68 years or 30-49 years (compared to 17 - 29 years); or in two of the ten training groups. There was no evidence that knowledge, attitudes or stigma scores for pre-training, post-training and change from pre to post were associated with risk of missing data at 6-months, therefore a complete case analysis comparing pre-test and 6-month follow-up scores is unlikely to be biased.

### Results of outcomes assessed

#### Knowledge of mental illnesses

As a preliminary step, inter-rater reliability was assessed. For the depression vignette, kappa for the correct response 'depression' was 0.94. For the schizophrenia vignette, kappas for the correct responses of 'schizophrenia' and 'psychosis' were 0.96 and 0.92 respectively.

There was strong evidence (p < 0.001) of an improvement in participants' knowledge about mental health problems from pre to post training. The mean knowledge score (out of 21) increased from 11.44 (SD = 3.23) pre-training to 15.86 (SD = 2.63) post training (mean difference 4.42, 95% CI 4.0, 4.83). There was a significant improvement in the proportion of participants answering correctly for 19 out of the 21 individual items.

For participants with follow-up data, the difference continued to be significant (p < 0.001), with an increase in mean score from 11.96 (SD = 3.16) pre-training to 15.15 (SD = 2.47) at follow-up (mean difference 3.19, 95% CI 2.63, 3.75).

#### Recognition of mental illnesses

Prior to undertaking YMHFA training, 89.6% of participants correctly recognised the mental illness described in the depression vignette. Post-training, 91.4% of participants correctly identified depression, however, the difference was not significant. For those with 6-month follow-up data, there was a significant improvement from pre-test to follow-up (from 89.1% to 97.1%, p = 0.019).

Pre-training, 67.4% of participants correctly recognised the illness in the schizophrenia vignette, and this increased to 91.8% post-training (p < 0.001). For those with 6-month follow-up data, there was a significant improvement from pre-test to follow-up (from 68.8% to 83.3%, p = 0.005).

#### Confidence in providing MHFA

For both vignettes, there was strong evidence of improvement in participants' confidence in helping a young person with a mental health problem. Pre-training, 31.8% reported feeling 'quite a bit' or 'extremely' confident in helping the young person described in the depression vignette; this increased to 84.1% post training (p < 0.001). Pre-training, 12.4% of participants reported feeling 'quite a bit' or 'extremely' confident in helping the young person described in the schizophrenia vignette; this increased to 60.8% post-training (p < 0.001).

For those with 6-month follow-up data, there was a significant improvement from pre-test to follow-up in confidence for both depression (from 30.8% to 72.2%, p < 0.001) and schizophrenia (from 11.8% to 40.4%, p < 0.001).

#### First aid intentions: knowledge of mental health first aid action plan

As a preliminary step, agreement was assessed between the person who rated all responses and a consensus rating of a sub-set of responses. The correlation between the consensus score and the overall rater for each action was: A = 0.98, L = 0.89, G = 0.80, E1 = 0.76, E2 = 0.87, Total = 0.96.

There was strong evidence of improvement in participants' knowledge of appropriate mental health first aid actions for both depression (p < 0.001) and schizophrenia (p < 0.001). For depression, the mean total ALGEE score prior to training was 3.13 (SD = 1.34) and increased to 5.57 (SD = 2.32) post-training. For schizophrenia, the mean total ALGEE score increased from 2.26 (SD = 1.19) at pre-training to 4.76 (SD = 2.11) post-training. A significant increase in the score from pre- to post-training occurred for each of the individual recommended actions for both depression and schizophrenia (p ≤ 0.006), except for recommending professional help for depression (p = 0.113).

For those with 6-month follow-up data, there was a significant improvement from pre-test to follow-up in knowledge of the MHFA Action Plan for both depression (from 3.25(SD = 1.30) to 3.95 (SD = 2.15), p = 0.043) and schizophrenia (from 2.27 (SD = 1.21) to 3.39 (SD = 2.02), p < 0.001).

#### Stigmatising attitudes about mental health problems

As shown in Tables 1 and 2, many participants disagreed with stigmatising attitudes towards depression and schizophrenia prior to training, however, there were still improvements after the training was completed.

**Table 1:** Personal disagreement with stigmatising attitudes about depression pre- and post-training

		Percentage 'disagree'/'strongly disagree'		
	**n**	**Pre-training %**	**Post-training %**	**p-value**

People with problems like Jenny could snap out of it if they wanted to	218	88.1	94.5	0.007
A problem like Jenny's is a sign of personal weakness.	219	94.1	98.2	0.026
Jenny's problem is not a real medical illness.	219	90.9	95.0	0.108
People with a problem like Jenny's are dangerous.	218	65.1	75.7	0.004
It is best to avoid people with a problem like Jenny's so that you don't develop this problem.	219	99.1	99.5	1.0
People with a problem like Jenny's are unpredictable.	219	35.2	52.1	<0.001
If I had a problem like Jenny's, I would not tell anyone.	218	72.9	78.4	0.119

**Table 2:** Personal disagreement with stigmatising attitudes about schizophrenia pre- and post-training

		Percentage 'disagree'/'strongly disagree'		
	**n**	**Pre-training %**	**Post-training %**	**p-value**

People with problems like John could snap out of it if they wanted to	221	94.6	97.3	0.07
A problem like John's is a sign of personal weakness.	220	95.0	98.6	0.022
John's problem is not a real medical illness.	220	93.2	95.9	0.146
People with a problem like John's are dangerous.	221	37.6	49.8	0.005
It is best to avoid people with a problem like John's so that you don't develop this problem.	220	96.4	98.6	0.18
People with a problem like John's are unpredictable.	221	20.4	33.0	<0.001
If I had a problem like John's, I would not tell anyone.	221	58.4	68.8	0.0052

For the group with 6-month follow-up data, the changes in stigmatising attitudes about depression from pre-test to follow-up showed the same patterns, except that the change in belief in dangerousness was no longer significant (64.2% at pre-test vs 72.3% at follow-up, p = 0.117). For the group with 6-month follow-up data, the changes in stigmatising attitudes about schizophrenia from pre-test to follow-up were only significant for disagreement about dangerousness (from 33.1% to 48.5%, p = 0.008).

The results for perceived stigma showed that participants believed that 'most other people' agreed with many of the stigmatising attitudes expressed in the statements shown. There was little change in participants' perceived stigma post-training.

#### Mental Health First Aid actions taken

There was a significant increase in the reported frequency of talking to a young person about their mental health problem compared to before training. Pre-training, 75.2% of participants reported talking to a young person about their mental health problem once or more in the previous six months, compared with 88.4% of participants at 6-month follow-up (p = 0.003).

Table 3 shows the strategies used by participants who talked to a young person about their mental health problem in the preceding six months. There was a significant increase in recommending professional help, recommending self-help strategies, giving information about the young person's problem and about local services. There was no significant change in participants' reporting that they had talked to the young person about suicidal thoughts. While a significant improvement in referral to books or websites was detected, the percentage of participants who reported doing this 6-months post-training was quite low.

**Table 3:** Strategies employed in talking to a young person about their mental health problem in the past 6 months

	Pre-training (n = 99)	6-months (n = 99)	p-value
*Strategies employed (frequency ≥ 'Once'):*			
Spent time listening to their problem	91.9%	99%	0.016
Helped to calm them down	66.7%	66.7%	-*
Talked to them about suicidal thoughts	46.5%	54.6%	0.169
Recommended they seek professional help	72.7%	87.9%	0.003
Recommended self-help strategies	45.5%	68.7%	<0.001
Gave them information about their problem	19.2%	45.5%	< 0.001
Gave them information about local services	44.4%	63.6%	0.002
Made an appointment for them with services	31.3%	37.4%	0.327
Referred them to books or websites about their problem	20.2%	32.3%	0.043

* could not be estimated.

### Program dissemination

Instructor training began in April, 2007. Between then and June 2010, 318 Youth Mental Health First Aid instructors were trained, of whom 274 remain accredited. There are instructors in every state and territory of Australia. A total of 872 completed courses have been registered, for a total of 10,686 participants. This result reflects a high level of interest in the program, and indicates that many individuals and organisations see the program as relevant and valuable.

Organisations which have funded their own staff to become YMHFA trainers include state education departments, welfare services (both government and non-government) and government health services. In addition, many individuals have funded themselves to become instructors and deliver courses for interested members of the public and on a consultancy basis to other organisations.

YMHFA was originally rolled out with funding provided by the Australian Government's National Suicide Prevention Strategy. This funding provided a salary for the original program co-ordinator (CS) and a number of scholarships for instructor training. YHMFA now sustains itself by charging fees for instructors to be trained.

In 2008, Mental Health First Aid Canada launched their youth program, with a focus on young people aged 15-25 years old [18]. Mental Health First Aid Singapore launched their youth program early in 2010 [19] and MHFA England is launching a YMHFA program in November 2010 [20]. Youth courses will start in Sweden and Hong Kong [21] and mainland China in 2011.

## Discussion

### Results of the trials

Two trials have now been run to test the effectiveness of YMHFA; the uncontrolled trial reported here, and a randomised controlled trial in schools of a modified and abbreviated version of the course [12].

In the uncontrolled trial, YMHFA training was associated with increased mental health knowledge, greater disagreement with stigmatising attitudes, increased confidence in helping a young person with a mental health problem and increased helping behaviour. An uncontrolled trial is a simple, cost-effective design that is useful for generating initial information prior to a larger trial. However, without a control group, changes observed cannot be definitively attributed to an intervention rather than other factors which could have influenced participants. Repeating the questionnaire, might in itself, lead to changed scores. In this study, high loss to follow-up occurred after 6 months, however the analysis undertaken suggests that missing data was unlikely to contribute bias to effects detected. Prior to training, participants reported high levels of personal experience of mental health problems (in self and family); this group may have had greater personal exposure to, or awareness of, mental health problems, and in this respect, might not be representative of the wider Australian adult population. However, the sample might be somewhat representative of other populations of interest, for example, adults with experience in relating to young people and an interest in mental health.

Participants in the school-based randomised controlled trial increased participants' knowledge, changed beliefs about treatment to be more like those of mental health professionals, reduced some aspects of stigma towards people with mental health problems, and increased confidence in offering help to students or colleagues. There was an indirect effect on students, who reported receiving more information about mental health from school staff. Most of the changes were sustained 6 months after training. However, no effects were found on participants' individual support towards students or on student mental health. While the randomised controlled trial was methodologically superior, it was not conducted using the full 14-hour program. The program delivered was an abbreviated course, with only senior school staff and those with welfare responsibilities receiving information about assisting in a crisis, and was also altered to include a component about school policy.

Although it is not possible to directly compare the results of the two trials, the full 14-hour course may produce greater improvements overall.

### Ongoing improvements to Youth Mental Health First Aid

The above evaluations were carried out using the first edition of the YMHFA course. The course has since been further developed and updated.

Between 2005 and 2008, a number of mental health first aid guidelines projects were carried out [22-30] to give a firmer basis to the content of MHFA training. Guidelines were developed for a number of developing disorders (depression, psychotic illnesses, eating disorders, alcohol use problems and other drug use problems), and a number of crisis situations (suicidal thoughts and behaviours, non-suicidal self-injury, panic attacks, traumatic events, severe psychosis, the acute effects of alcohol and other drug use including medical emergencies, and apparent aggressive behaviour). These projects used the Delphi method, a technique for reaching consensus within and between groups of experts. The panels of experts were professionals (clinicians and researchers), people with experience of mental illness, and care-givers of people with mental illness. Both the youth and standard courses and course manuals have been thoroughly updated using the content of the new guidelines [31,32].

Some text changes have been made to the actions 1, 3 and 5 of the MHFA Action Plan. The acronym is still 'ALGEE', but the actions are now as follows:

• Approach, assess and assist with any crisis

• Listen non-judgmentally

• Give support and information

• Encourage the person to get appropriate professional help

• Encourage other supports

Other changes to the course content have been made based on ongoing feedback from instructors and participants as recorded on the MHFA website. For example, the original YMHFA materials did not discuss the difference between how a parent might apply MHFA and how another adult might, nor about how another adult might need to involve parents in the process. Other improvements to the curriculum include considerations of how to address communication difficulties and a greater focus on skills than knowledge.

The updated course is being described as Edition 2. In 2010, every accredited instructor has had to attend a one-day update workshop orienting them to the Edition 2 materials. From 2011, only Edition 2 may be taught. Initial feedback from instructors on the new materials has been very positive.

### Aims for the future

Future target groups for YMHFA training include juvenile justice, police and other emergency services. In addition, we hope to encourage all Australian schools to make training available to all school staff. Another group which is an ideal target for training is parents, because parents are often the first people that an adolescent will turn to when they are concerned about their own mental health [33].

The next evaluation planned, using the Edition 2 materials, is a randomised controlled trial of parents of adolescents. Our intention is to follow up the families for a number of years to see if the course does improve early identification of mental health problems and prompt help-seeking. The advantage of this sort of evaluation is that it enables us to test the outcome for people who receive Mental Health First Aid, a group which has previously been difficult to follow up.

## Conclusions

The Youth Mental Health First Aid course provides participants with the knowledge and skills needed to assist a young person who is experiencing a mental health problem. The results of evaluation studies and expert consensus guidelines have been used to further refine and improve the curriculum. The course appears to be valued and relevant to many sectors of the public and has been adopted across Australia and in a number of other countries.

## Competing interests

A number of the authors were involved in the development and roll out of the YMHFA course.

## Authors' contributions

CMK had primary responsibility for writing the manuscript. JMM conducted statistical analysis on the uncontrolled trial and contributed to the writing of the results section of the manuscript. JAF was responsible for the 6-month follow-up in the uncontrolled trial, data entry, data quality and scoring of open-ended data, and contributed to the writing of the manuscript. BAK contributed to the development of the YMHFA course and the design of the evaluation. AFJ had primary responsibility for the design of the evaluation, and contributed to both the data analysis and the writing of the manuscript. AL contributed to the statistical analysis of the uncontrolled trial. CS taught all the courses in the uncontrolled trial and gathered the pre-test and post-test data. All authors read and approved the final manuscript.

## Supplementary Material

Additional file 1 **Questionnaire (PDF)**. This is the pre-test questionnaire used before the intervention. For the questionnaire used post-test, the sociodemographics (questions 1 to 10) were omitted along with the actions taken in the last six months (question 22). For the 6-month follow-up, the sociodemographics were omitted but the actions taken in the last six months were measured again. Click here for file
