# Supplementary material for: Youth mental health first aid: a description of the program and an initial evaluation
Source: Int J Ment Health Syst. 2011 Jan 27;5:4. doi: 10.1186/1752-4458-5-4 (PMC3041764; doi:10.1186/1752-4458-5-4)
Supplement: Additional file 1 — Questionnaire (PDF). This is the pre-test questionnaire used before the intervention. For the questionnaire used post-test, the sociodemographics (questions 1 to 10) were omitted along with the actions taken in the last six months (question 22). For the 6-month follow-up, the sociodemographics were omitted but the actions taken in the last six months were measured again. [file 1752-4458-5-4-S1.PDF]

PRE-COURSE QUESTIONNAIRE

YOUTH MENTAL HEALTH FIRST AID

You are taking part in a Youth Mental Health First Aid course run by the ORYGEN Research Centre. We wish to find out how useful this course is in providing skills to help young people with a mental health problem.

For this reason, we are asking all participants to fill out a questionnaire at **the beginning** and end of the course and 6 months after the course.

Of course, completing this questionnaire is voluntary. However, we hope you will understand our reasons for seeking this information and you will help by filling out this questionnaire.

All information will be kept confidential and will only be used to give summary statistics of the course.

Thank you

Chris Scanlan
Youth Mental Health First Aid Coordinator

ID ☐☐☐

QUESTIONNAIRE

EVALUATION OF THE YOUTH MENTAL HEALTH FIRST AID COURSE

1. What gender are you?

   1 ☐ Male
   2 ☐ Female

2. How old are you?

   ☐ ☐ years

3. What is the highest level of schooling that you completed?

   1 ☐ Some Primary
   2 ☐ All Primary
   3 ☐ Some secondary
   4 ☐ Three/four years of secondary (intermediate or school certificate level)
   5 ☐ Five/six years of secondary (leaving or higher school certificate level)

4. What is the highest level of post secondary/tertiary education you completed?

   1 ☐ Trade/apprenticeship
   2 ☐ Other certificate
   3 ☐ Associate or undergraduate diploma
   4 ☐ Bachelor's degree
   5 ☐ Higher degree
   6 ☐ Other
   7 ☐ None

5. Are you presently studying for any of the following?

   1 ☐ Trade/apprenticeship
   2 ☐ Other certificate
   3 ☐ Associate or undergraduate
   4 ☐ Diploma
   5 ☐ Bachelor's Degree
   6 ☐ Higher degree
   7 ☐ Other
   8 ☐ None

6. Are you any of the following:(tick as many as necessary)

   1 ☐ Aboriginal
   2 ☐ Torres Strait Islander
   3 ☐ South Sea Islander
   4 ☐ Refugee/Migrant
   5 ☐ Australian
   6 ☐ Other

7. Do you speak a language other than English as your first language at home?

   1 ☐ Yes
   2 ☐ No

8. Why are you interested in doing this course?

____

____

____

____

____

9. Have you ever done any previous mental health training?

1 ☐ Yes
2 ☐ No

If yes, please describe this training:

____

____

____

10. We would like to know a bit about your awareness and experience of mental health problems in everyday life.

a) Have you yourself <u>ever</u> experienced a mental health problem?

1 ☐ Yes
2 ☐ No

b) Has anyone in your family <u>ever</u> experienced a mental health problem?

1 ☐ Yes
2 ☐ No

The following section concerns a hypothetical young person called Jenny. The description below outlines how she has been recently.

Jenny is a 15 year old who has been feeling unusually sad and miserable for the last few weeks. She is tired all the time and has trouble sleeping at night. Jenny doesn't feel like eating and has lost weight. She can't keep her mind on her studies and her marks have dropped. She puts off making any decisions and even day-to-day tasks seem too much for her. Her parents and friends are very concerned about her. Jenny feels she will never be happy again and believes her family would be better off without her. She has been so desperate, she has been thinking of ways to end her life.

11. What, if anything, do you think is wrong with Jenny?

____

____

____

12. Imagine Jenny is a young person you know. You want to help her. What would you do?

____

____

____

____

____

____

13. How confident would you feel in helping Jenny?

1 ☐ Not at all
2 ☐ A little bit
3 ☐ Moderately
4 ☐ Quite a bit
5 ☐ Extremely

The next few questions contain statements about Jenny's problem. Please indicate how strongly YOU PERSONALLY agree or disagree with each statement.

14.

		Strongly agree	*Agree*	*Neither agree nor disagree*	*Disagree*	*Strongly disagree*
a)	People with problems like Jenny could snap out of it if they wanted to	1☐	2☐	3☐	4☐	5☐
b)	A problem like Jenny's is a sign of personal weakness.	1☐	2☐	3☐	4☐	5☐
c)	Jenny's problem is not a real medical illness.	1☐	2☐	3☐	4☐	5☐
d)	People with a problem like Jenny's are dangerous.	1☐	2☐	3☐	4☐	5☐
e)	It is best to avoid people with a problem like Jenny's so that you don't develop this problem.	1☐	2☐	3☐	4☐	5☐
f)	People with a problem like Jenny's are unpredictable.	1☐	2☐	3☐	4☐	5☐
g)	If I had a problem like Jenny's, I would not tell anyone.	1☐	2☐	3☐	4☐	5☐

Now we would like you to tell us what you think MOST OTHER PEOPLE believe. Please indicate how strongly you agree or disagree with the following statements.

15.

		Strongly agree	*Agree*	*Neither agree nor disagree*	*Disagree*	*Strongly disagree*
a)	Most other people believe that people with a problem like Jenny's could snap out of it if they wanted.	1☐	2☐	3☐	4☐	5☐
b)	Most people believe that a problem like Jenny's is a sign of personal weakness.	1☐	2☐	3☐	4☐	5☐
c)	Most people believe that Jenny's problem is not a real medical illness.	1☐	2☐	3☐	4☐	5☐
d)	Most people believe that people with a problem like Jenny's are dangerous.	1☐	2☐	3☐	4☐	5☐
e)	Most people believe that it is best to avoid people with a problem like Jenny's so that you don't develop this problem.	1☐	2☐	3☐	4☐	5☐
f)	Most people believe that people with a problem like Jenny's are unpredictable.	1☐	2☐	3☐	4☐	5☐
g)	If they had a problem like Jenny's, most people would not tell anyone.	1☐	2☐	3☐	4☐	5☐

The following section concerns a hypothetical young person called John. The description below outlines how he has been recently.

John is a 15 year old who lives at home with his parents. He has been attending school irregularly over the past year and has recently stopped attending altogether. Over the past six months he has stopped seeing his friends and begun locking himself in his bedroom and refusing to eat with the family or to have a bath. His parents also hear him walking about in his bedroom at night while they are in bed. Even though they know he is alone, they have heard him shouting and arguing as if someone else is there. When they try to encourage him to do more things, he whispers that he won't leave home because he is being spied upon by the neighbour. They realize he is not taking drugs because he never sees anyone or goes anywhere.

16. What, if anything, do you think is wrong with John?

____

____

____

17. Imagine John is a young person you know. You want to help him. What would you do?

____

____

____

____

____

____

18. How confident would you feel in helping John?

1 ☐ Not at all
2 ☐ A little bit
3 ☐ Moderately
4 ☐ Quite a bit
5 ☐ Extremely

The next few questions contain statements about John's problem. Please indicate how strongly YOU PERSONALLY agree or disagree with each statement.

19.

	Strongly agree	*Agree*	*Neither agree nor disagree*	*Disagree*	*Strongly disagree*
a) People with problems like John could snap out of it if they wanted to	1☐	2☐	3☐	4☐	5☐
b) A problem like John's is a sign of personal weakness.	1☐	2☐	3☐	4☐	5☐
c) John's problem is not a real medical illness.	1☐	2☐	3☐	4☐	5☐
d) People with a problem like John's are dangerous.	1☐	2☐	3☐	4☐	5☐
e) It is best to avoid people with a problem like John's so that you don't develop this problem.	1☐	2☐	3☐	4☐	5☐
f) People with a problem like John's are unpredictable.	1☐	2☐	3☐	4☐	5☐
g) If I had a problem like John's, I would not tell anyone.	1☐	2☐	3☐	4☐	5☐

Now we would like you to tell us what you think MOST OTHER PEOPLE believe. Please indicate how strongly you agree or disagree with the following statements.

20.

	Strongly agree	*Agree*	*Neither agree nor disagree*	*Disagree*	*Strongly disagree*
a) People with problems like John could snap out of it if they wanted to	1☐	2☐	3☐	4☐	5☐
b) A problem like John's is a sign of personal weakness.	1☐	2☐	3☐	4☐	5☐
c) John's problem is not a real medical illness.	1☐	2☐	3☐	4☐	5☐
d) People with a problem like John's are dangerous.	1☐	2☐	3☐	4☐	5☐
e) It is best to avoid people with a problem like John's so that you don't develop this problem.	1☐	2☐	3☐	4☐	5☐
f) People with a problem like John's are unpredictable.	1☐	2☐	3☐	4☐	5☐
g) If I had a problem like John's, I would not tell anyone.	1☐	2☐	3☐	4☐	5☐

<u>**OPINIONS ABOUT MENTAL HEALTH PROBLEMS**</u>

For each of the statements below please indicate whether you agree or disagree with it, or don't know.

21.

		Agree	*Disagree*	*Don't know*
a)	Most adolescents with mental health problems get some sort of professional help.	1☐	2☐	3☐
b)	Around half of mental disorders start during childhood or adolescence	1☐	2☐	3☐
c)	Schizophrenia is one of the most common mental disorders in Australia.	1☐	2☐	3☐
d)	It is not a good idea to ask someone if they are feeling suicidal in case you put the idea in their head.	1☐	2☐	3☐
e)	Depression can increase a young person's risk taking behaviour, e.g. reckless driving, risky sexual involvements.	1☐	2☐	3☐
f)	Antidepressants should be the first line of treatment for depressed adolescents.	1☐	2☐	3☐
g)	If someone has a traumatic experience, it is best to make them talk about it as soon as possible.	1☐	2☐	3☐
h)	It is best to get someone having a panic attack to slow down their breathing.	1☐	2☐	3☐
i)	Anxiety disorders in adolescence increase the risk for a range of other mental disorders in adulthood.	1☐	2☐	3☐
j)	People who develop psychosis have often had a poor relationship with their mother.	1☐	2☐	3☐
k)	It is best not to try to reason with people having delusions.	1☐	2☐	3☐
l)	People with a psychosis tend to have a better outcome if family members are not critical of them.	1☐	2☐	3☐
m)	Smoking is much more common in adolescents with depression.	1☐	2☐	3☐
n)	Cannabis is the substance of abuse most commonly used by adolescents.	1☐	2☐	3☐
o)	If a person has overdosed and is still conscious, do not give them anything to drink.	1☐	2☐	3☐
p)	The most common type of eating disorder is anorexia nervosa.	1☐	2☐	3☐
q)	When talking to people with eating disorders, it is important not to criticize their body size.	1☐	2☐	3☐
r)	The best treatment for eating disorders is medication.	1☐	2☐	3☐
s)	People who harm themselves nearly always want to die.	1☐	2☐	3☐
t)	Self-harm can be used to help escape from negative feelings such as hopelessness.	1☐	2☐	3☐
u)	Most people who self-harm are seeking attention.	1☐	2☐	3☐

Over the past 6 months:

22.

a) Have you talked to a young person about their mental health problem?

1 ☐ Never
2 ☐ Once
3 ☐ A few times
4 ☐ Many times

b) If yes, did you do any of the following? (You can choose more than one):

1 ☐ Spent time listening to their problem
2 ☐ Helped to calm them down
3 ☐ Talked to them about suicidal thoughts
4 ☐ Recommended they seek professional help
5 ☐ Recommended self-help strategies
6 ☐ Gave them information about their problem
7 ☐ Gave them information about local services
8 ☐ Made an appointment for them with services
9 ☐ Referred them to books or websites about their problem

c) Did you do anything else?

____

____

____

____

____

____

EVALUATION OF THE YOUTH MENTAL HEALTH FIRST AID COURSE

TEAR OFF SHEET

Please note that we require these details in order to send you the questionnaire for follow up at 6 months. These details will be stored separately to the rest of the questionnaire.

Name: ………………………………………………

Postal Address:
………………………………………………………………………………………………
………………………………………………………………………………………………
………………………………………………………………………

Post-code: ………..

Phone: ……………………………..

Email: ……………………………………………………………

Assigned Identification Number: ☐☐☐
